# _D_-Glutamate is metabolized in the heart mitochondria

**DOI:** 10.1038/srep43911

**Published:** 2017-03-07

**Authors:** Makoto Ariyoshi, Masumi Katane, Kenji Hamase, Yurika Miyoshi, Maiko Nakane, Atsushi Hoshino, Yoshifumi Okawa, Yuichiro Mita, Satoshi Kaimoto, Motoki Uchihashi, Kuniyoshi Fukai, Kazunori Ono, Syuhei Tateishi, Daichi Hato, Ryoetsu Yamanaka, Sakiko Honda, Yohei Fushimura, Eri Iwai-Kanai, Naotada Ishihara, Masashi Mita, Hiroshi Homma, Satoaki Matoba

**Affiliations:** 1 Department of Cardiovascular Medicine, Graduate School of Medical Science, Kyoto Prefectural University of Medicine, Kawaramachi-Hirokoji, Kamigyo-ku, Kyoto 602-8566, Japan; 2 Laboratory of Biomolecular Science, Graduate School of Pharmaceutical Sciences, Kitasato University, 5-9-1 Shirokane, Minato-ku, Tokyo 108-8641, Japan; 3 Graduate School of Pharmaceutical Sciences, Kyushu University, 3-1-1 Maidashi, Higashi-ku, Fukuoka 812-8582, Japan; 4 Shiseido Co., Ltd., 1-1-16 Higashi-shimbashi, Minato-ku, Tokyo 105-0021, Japan; 5 Faculty of Health Care, Tenri Health Care University, 80-1 Bessho-cho, Tenri, Nara 632-0018, Japan; 6 Department of Protein Biochemistry, Institute of Life Science, Kurume University, 1-1 Hyakunen-koen, Kurume 839-0864, Japan

## Abstract

_D_-Amino acids are enantiomers of L-amino acids and have recently been recognized as biomarkers and bioactive substances in mammals, including humans. In the present study, we investigated functions of the novel mammalian mitochondrial protein 9030617O03Rik and showed decreased expression under conditions of heart failure. Genomic sequence analyses showed partial homology with a bacterial aspartate/glutamate/hydantoin racemase. Subsequent determinations of all free amino acid concentrations in 9030617O03Rik-deficient mice showed high accumulations of D-glutamate in heart tissues. This is the first time that a significant amount of D-glutamate was detected in mammalian tissue. Further analysis of D-glutamate metabolism indicated that 9030617O03Rik is a D-glutamate cyclase that converts D-glutamate to 5-oxo-D-proline. Hence, this protein is the first identified enzyme responsible for mammalian D-glutamate metabolism, as confirmed in cloning analyses. These findings suggest that D-glutamate and 5-oxo-D-proline have bioactivities in mammals through the metabolism by D-glutamate cyclase.

The majority of amino acids in higher animals are thought to be L-enantiomers and _D_-amino acid enantiomers are considered unnatural. Moreover, _D_-amino acids are indispensable for bacterial growth as components of cell wall peptidoglycans. However, advanced analytical techniques have demonstrated that several _D_-amino acids are present in mammals, including humans^1^,^2^. Accordingly, _D_-amino acids have attracted recent attention as candidate biomarkers and novel bioactive substances^3^. Moreover, physiologic functions of several _D_-amino acids have been identified to date. In particular, D-serine regulates nervous signaling in the cerebral cortex and participates in memorization and learning^4^,^5^. D-Aspartate is particularly present in central nervous, neuroendocrine, and endocrine systems and plays physiological roles in the regulation of hormone secretion and steroidogenesis^6^,^7^.

In the present study, we investigated a novel mitochondrial protein with a currently unknown function. Specifically, expression of the protein 9030617O03Rik was decreased under conditions of heart failure, although high expression was identified in the heart, kidney, and liver tissues of healthy mice. Subsequent mitochondrial localization experiments showed that this protein is present in the internal matrix membrane. In addition, enzyme assays indicated for the first time that this mitochondrial protein possesses D-glutamate cyclase activity that converts D-glutamate to 5-oxo-D-proline. A significant amount of D-glutamate in mammalian tissues has not been reported yet. In the present study, we utilized a two-dimensional high-performance liquid chromatography (2D-HPLC) combined with highly sensitive fluorescence derivation, thereby being the first study to achieve detection of D-glutamate at a high level in the hearts of 9030617O03Rik-deficient mice. The present study suggests that additional research would reveal the roles of D-glutamate and D-glutamate cyclase in mammals.

## Results

### 9030617O03Rik is reduced during pressure overload heart failure

**Figure 1:**
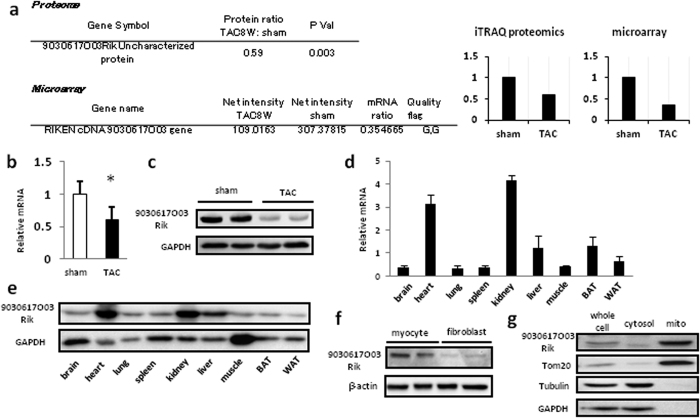
Mitochondrial 9030617O03Rik protein expression was decreased in transverse aortic constriction (TAC)-operated failing hearts. (**a**) Raw data from proteome and microarray. (**b,c**) 9030617O03Rik messenger RNA (mRNA) and protein expression in mouse TAC failing hearts were assessed using real time polymerase chain reaction (PCR) and immunoblotting, respectively. (**d**,**e**) Comparison of real-time reverse transcription (RT)-PCR analyses of 9030617O03Rik mRNA and immunoblot analyses of 9030617O03Rik protein between organs; 9030617O03Rik was predominantly expressed in heart, kidney, and liver tissues BAT, brown adipose tissue WAT, white adipose tissue. (**f**) Proportions of 9030617O03Rik protein in myocytes and fibroblasts from rat neonatal hearts were evaluated using immunoblotting. (**g**) Localization of 9030617O03Rik protein in rat neonatal myocytes was evaluated using immunoblotting with Tom20 as a marker for mitochondrial proteins. Tubulin and glyceraldehyde 3-phosphate dehydrogenase (GAPDH) were used as markers for cytosolic proteins. Data are shown as the means ± standard deviation(S.D.). *p < 0.01.

To detect novel mammalian mitochondrial molecules that regulate the progression of heart failure, we compared protein expression between transverse aortic constriction (TAC)-operated hearts and sham-operated hearts from mice using mitochondrial proteome assays^8^. Heart mitochondria were purified and analyses with isobaric Tags for Relative and Absolute Quantitation (iTRAQ) were performed to enable simultaneous identification and relative quantification of mitochondrial proteins through isobaric peptide tagging. A total of 7,925 iTRAQ-labeled peptides were mapped to a total of 464 proteins, which were then identified and quantified in purified heart mitochondria. Among these, protein expression of 9030617O03Rik, which has not been functionally characterized, was decreased during heart failure. Moreover, an additional microarray comparison between the TAC group and the sham group showed that the messenger RNA (mRNA) of 9030617O03Rik was decreased (Figs 1a and S1). Both analyses were performed at Filgen (Nagoya, Japan). In addition, the decrease of 9030617O03Rik was confirmed by real-time polymerase chain reaction (PCR) (Fig. 1b) and western blotting (Fig. 1c). Further analyses showed high expressions of 9030617O03Rik mRNA and protein in heart, kidney, and liver tissues (Fig. 1d,e), and protein structure predictions from a database showed that 9030617O03Rik comprises a single coiled-coil domain and two domains of unknown function (DUF).

### 9030617O03Rik is located in the mitochondrial matrix

**Figure 2:**
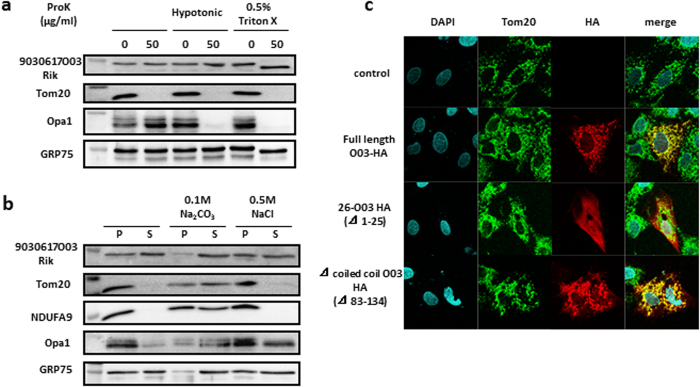
9030617O03Rik was located in the mitochondrial matrix and was attached to inner membrane. (**a**) (Pro K) protection assays show target protein locations in mitochondria. Isolated mouse liver mitochondria were incubated in hypotonic buffer or with Triton-X and were then treated with Proteinase K. Samples were then subjected to immunoblotting using antibodies against 9030617O03Rik or individual proteins. Tom20, Opa1, and GRP75 were used as markers of the outer membrane, inner membrane, and matrix, respectively. (**b**) Alkali/Salt extraction assays show strengths of target protein attachment to membranes. Isolated mouse liver mitochondria samples were subjected to 0.1 M Na_2_CO_3_ or 0.5 M NaCl and were divided into pellets (P) and supernatant (S). (**c**) Representative images of H9C2 rat cardiomyoblast cells transfected with various plasmids; cells were stained with anti- 4′,6-diamidino-2-phenylindole (DAPI) (blue) to visualize nuclei, anti-Tom20 (green) to visualize mitochondria, and anti-hemagglutinin (HA) (red) to visualize the expressed proteins; original magnification, x1,000.

To examine cardiac localization, we separated myocytes and fibroblasts from rat neonatal hearts and showed that 9030617O03Rik is almost exclusively present in cardiomyocytes (Fig. 1f). Cell fractionation analyses of rat hearts showed that 9030617O03Rik is located in mitochondria (Fig. 1g). Proteinase K protection assays (Fig. 2a) and alkali/salt extraction assays (Fig. 2b)^9^ were confirmed that 9030617O03Rik is a mitochondrial matrix protein of the inner membrane. Moreover, co-localization between 9030617O03Rik and mitochondria was demonstrated in immunofluorescence staining experiments following infection of rat H9C2 cardiomyoblast cells with various fragmented 9030617O03Rik sequences in influenza hemagglutinin (HA)-tagged plasmids. Full-length and coiled-coil domain deleted plasmids were co-localized with mitochondria, whereas the 1–25 deleted plasmid was not (Fig. 2c), indicating that N-terminal localization signals of 9030617O03Rik guide the molecule to mitochondria. In further studies, *in situ* hybridization of 9030617O03Rik in embryonic day 18.5 (e18.5d) mouse embryos demonstrated heart and kidney expression (Fig. S2a,b). In these experiments, 9030617O03Rik was expressed as early as e14.5d, and its expression was increased at least until e18.5d (Fig. S1c).

### Regions of DUF 4392 sequence homology with the aspartate/glutamate/hydantoin racemase of *Thermovirga lienii*

**Figure 3:**
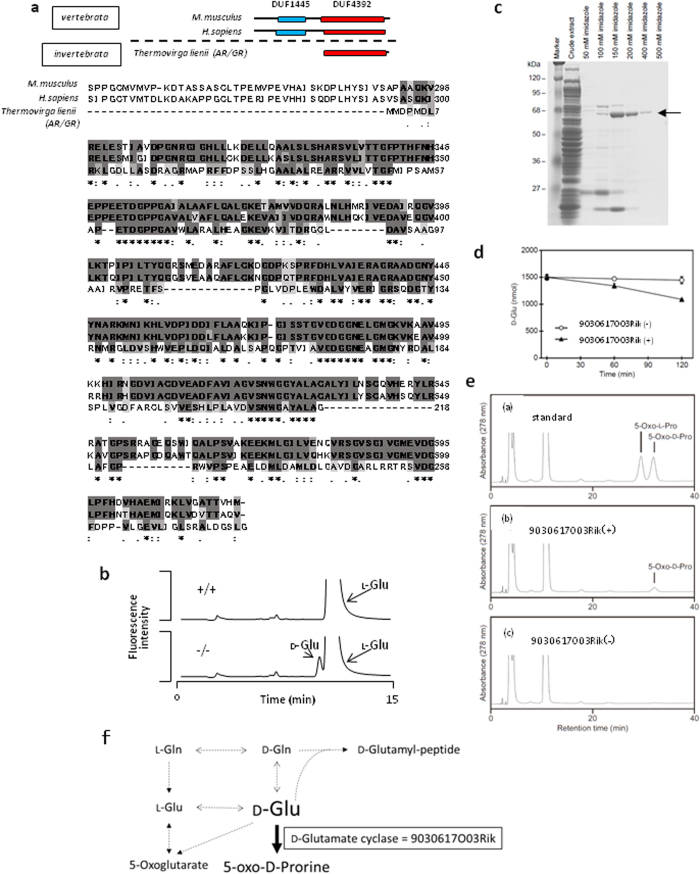
The mitochondrial 9030617O03Rik protein has D-glutamate cyclase activity and D-glutamate accumulates in the hearts of 9030617O03Rik systemic deficient mice. (**a**) A schematic representation of domain sequence comparisons between species; DUF 1445 and DUF 4392 were domains of unknown function that were identified among 9030617O03Rik sequences (upper). Amino acid alignments of 9030617O03Rik (mouse), c14orf159 (human homolog), and aspartate/glutamate/hydantoin racemase (*Thermovirga lienii*) (lower). These three species also have DUF4392. In addition, there is 32% homology between DUF4392 of 9030617O03Rik and *T. lienii* (below). “*” indicates positions which have a single, fully conserved residue. “:” indicates that one of the following “strong” groups is fully conserved:- STA, NEQK, NHQK, NDEQ, QHRK, MILV, MILF, HY, FYW. “.” indicates that one of the following “weaker” groups is fully conserved:- CSA, ATV, SAG, STNK, STPA, SGND, SNDEQK, NDEQHK, NEQHRK, FVLIM, HFY. These are all the positively scoring groups that occur in the Gonnet Pam250 matrix. The strong and weak groups are defined as strong score >0.5 and weak score ≤0.5, respectively. (**b**) Chromatograms from two-dimensional high-performance liquid chromatography (2D-HPLC) analyses of D-glutamate and L-glutamate. (**c**) Purity of recombinant 9030617O03Rik proteins after elution form a stepwise imidazole gradient. Purity and homogeneity of recombinant proteins were confirmed using sodium dodecyl sulfate (SDS)-polyacrylamide gel electrophoresis (PAGE). (**d**) Decreases in D-glutamate levels in the enzymatic reaction mixture with purified 9030617O03Rik protein were greater than in the control. (**e**) Chromatograms of standard 5-oxo-D- and 5-oxo-L-proline (a), sample treated with 9030617O03Rik protein (b), and untreated sample (c). (**f**) Pathways of D-glutamate metabolism from the Kyoto Encyclopedia of Genes and Genomes (KEGG) database.

National Center for Biotechnology Information (NCBI) protein database searches showed that the latter domain of unknown function DUF 4392 has sequence homology with the aspartate/glutamate/hydantoin racemase of *T. lienii* (Fig. 3a). Moreover, whereas DUF 4392 was conserved among vertebrates and invertebrates, the former domain DUF 1445 was conserved only in vertebrates.

### D-Glutamate accumulates in 9030617O03Rik knockout (KO) mouse hearts

Racemases catalyze the racemic conversion of L- and _D_-amino acids and were associated with amino acid metabolism. Subsequently, all amino acid profiles in 9030617O03Rik-deficient mouse hearts and kidneys were separately analyzed for L- and D-enantiomers using 2D HPLC analyses. In these experiments, KO mice were acquired from the KO mouse project (KOMP), and KO embryonic stem (ES) cells were examined using southern blotting analyses to confirm gene modifications (Fig. S3a,b). In these mice, the 9030617O03Rik protein was completely defective in all organs (Fig. S3c,d), although no phenotypes of mortality, size, cardiac function, or mitochondrial morphology were observed under normal conditions (Fig. S4). Subsequently, all free amino acids were divided into L- and D-enantiomers and analyzed using a 2D HPLC technique as described in our previous studies^2^,^10^. These analyses revealed highly elevated D-glutamate levels in 9030617O03Rik-deficient mouse hearts in comparison with wild type (WT) mouse hearts (Fig. 3b, Table 1 and Supplemental Table 1).

### 9030617O03Rik has D-glutamate cyclase activity

To investigate roles of 9030617O03Rik in D-glutamate metabolism, we analyzed enzyme activity using cloned and purified 9030617O03Rik protein, as described in the Materials and Methods. Following the purification of recombinant 9030617O03Rik (Fig. 3c), D-glutamate degradation activity was confirmed (Fig. 3d). Subsequent searches of the Kyoto Encyclopedia of Genes and Genomes (KEGG) showed that candidate genes for D-glutamate degradation included glutamate racemase, D-glutamate oxidase, and D-glutamate cyclase. The detailed methods of each enzymatic activity assay are shown in the Supplemental Information. Subsequently, the *in vitro* enzyme assay showed no glutamate racemase activity or D-glutamate oxidase activity of the protein. Figure S5 showed that there was no peak of L-glutamate in the reaction product by HPLC. Moreover, D-glutamate oxidase activity was below the limit of quantification using a colorimetric method for 2-oxoglutaric acid production. In addition, determinations of 5-oxo-D-proline production showed that 9030617O03Rik is a D-glutamate cyclase (Fig. 3e,f); therefore, D-glutamate cyclase was identified in mammalian tissue for the first time.

We found that D-glutamate cyclase was decreased in the mouse heart failure model. Future analyses will reveal the important roles of D-glutamate, D-glutamate cyclase, and 5-oxo-D-proline in mammals.

## Discussion

The present study showed decreased expression of the mitochondrial protein 9030617O03Rik in the heart failure mouse model. Accumulation of D-glutamate in the heart of 9030617O03Rik-deficient mice suggests that D-glutamate is metabolized in mammalian hearts. Since this protein was present in mitochondria, our initial investigations were performed using immunoprecipitation analyses. However, due to limited protein binding to 9030617O03Rik, these analyses failed. Subsequently, genomic sequence analysis indicated sequence homology of 9030617O03Rik domains with those from a bacterial amino acid racemase. Only two mammalian amino acid racemases have been previously reported, including a serine racemase^11^,^12^,^13^ and an aspartate racemase^14^. Moreover, the DUF4392 domain of 9030617O03Rik was homologous to an aspartate/glutamate/hydantoin racemase of *T. lienii*, suggesting that this domain is involved in interconversion between L- and _D_-amino acids. In subsequent experiments, 9030617O03Rik deficiency in mice was not embryonically lethal, and no differences in body weights, heart sizes, or heart contraction were noted in comparison with WT mice. However, analysis of all free amino acids, including L- and D-enantiomers, in hearts and kidneys of 9030617O03Rik-deficient mice and control mice revealed high accumulation of D-glutamate, especially in hearts of 9030617O03Rik-deficient mice, whereas D-glutamate was not detectable in control mice. Moreover, D-alanine was accumulated in KO kidneys. To understand the reason for D-alanine accumulation in the kidney, further investigation of their excretion and metabolism is necessary. Since D-alanine is metabolized by _D_-amino acid oxidase (DAO)^15^, future examination using DAO KO mice is essential.

Prior to the early 1980s, it was accepted that free _D_-amino acids are not present in higher animals. However, technological advances have since enabled identification of various _D_-amino acids in humans and other mammals^1^,^16^,^17^. Among these, D-serine and D-aspartate are predominant in mammals, and following conversion from D-serine by serine racemases, D-serine reportedly acts as an endogenous ligand for the glutamate *N*-methyl-D-aspartate (NMDA) receptor^13^. Similarly, D-aspartate has been detected in mammalian brain, pineal gland, pituitary gland, adrenal gland, and testis tissues^18^,^19^, suggesting synthesis by aspartate racemase and degradation by D-aspartate oxidases (DDO), as well as important roles in the regulation of hormones.

It is generally accepted that D-glutamate is not produced in higher mammals, but is well known as a bacterial cell wall component. D-Glutamate is also present in foods such as soybeans and arises from the turnover of intestinal tract microflora, whose cell walls contain significant D-glutamate contents. Accordingly, exogenous D-glutamate is abundant in mammalian bodies, and although D-glutamate is a potent natural inhibitor of glutathione synthesis^20^, details of its metabolism in mammals remain undescribed. Unlike other D-amino acids, D-glutamate is not oxidized by DAO but may be subject to oxidation by DDO. However, DDO metabolism of D-glutamate has not yet been demonstrated *in vivo*, although previous studies have identified DDO in kidney, liver, and brain tissues^19^. Moreover, D-glutamate has been detected in blood^21^, saliva^22^, and urine^23^,^24^, and trace quantities have been detected in mammalian tissues^24^,^25^,^26^. The present data are the first to demonstrate significant quantities of D-glutamate in mammalian organs of 9030617O03Rik KO mice, especially in hearts. Thus, to further examine the association of 9030617O03Rik with D-glutamate metabolism, we investigated potential D-glutamate oxidase, glutamate racemase, D-glutamyltransferase, D-glutamate ligase, and D-glutamate cyclase activities and identified 9030617O03Rik as a D-glutamate cyclase.

In older studies, D-glutamate cyclase was purified from mammalian organs such as kidneys and livers, and enzymatic conversion of D-glutamate to 5-oxo-D-proline was reported^27^,^28^,^29^. However, the origin of D-glutamate was unknown in these studies, and it was also found that normal humans excreted 50–400 μmoles of 5-oxo-D-proline (and much less 5-oxo-L-proline) per day^29^. These studies suggested that exogenous D-glutamate is metabolized by dietary enzymes and bacterial flora. Accordingly, oral loading of D-glutamate in healthy human volunteers resulted in progressive increases in plasma 5-oxo-D-proline to levels that were ten-fold higher than plasma D-glutamate concentrations^30^. These reports also suggested the presence of a D-glutamate metabolism pathway in humans. However, no genes encoding such enzymes were identified. Hence, the present analyses are the first to demonstrate the presence of a mammalian D-glutamate cyclase gene, warranting further characterizations of D-glutamate cyclase and evaluations of the physiological roles of D-glutamate and 5-oxo-D-proline in mammals.

In summary, we demonstrated that 9030617O03Rik is a mammalian D-glutamate cyclase and that D-glutamate accumulates in heart tissues of D-glutamate cyclase deficient mice.

## Methods

All experiments were performed in accordance with Bioethics Committee of Kyoto Prefectural University of Medicine (approval number M25-121, 20-37). Further details of enzyme activity assays can be found in the Supplemental Materials and Methods.

### Mice experiments

All animal studies were approved by the Bioethics Committee of Kyoto Prefectural University of Medicine. Male mice with systemic 9030617O03Rik deficiency (C57BL6J background) were generated using a KO ES cell line from the KOMP Repository. TAC was induced in 8–10-week-old mice following intubation and ventilation with room air using a small animal respirator (SN-480-7, Shinano Seisakusyo, Tokyo, Japan), and 1.0% isoflurane inhalation was added to maintain anesthesia. The left side of the chest was opened at the second intercostal space, and aortic constriction was performed by ligation of the transverse thoracic aorta between the innominate artery and left common carotid artery using a 28 gauge needle and a 7-0 silk string. Sham operations were performed without constricting the aorta.

### Mitochondrial isolation

Mouse hearts were rapidly minced in ice cold MSE buffer containing 220 mM mannitol, 70 mM sucrose, 2 mM ethylene glycol-bis(β-aminoethyl ether)-N,N,N′,N′-tetraacetic acid (EGTA), 5 mM 3-(N-morpholino)propanesulfonic acid (MOPS) (pH 7.4), 2 mM taurine, and 0.2% bovine serum albumin (BSA). Heart tissues were homogenized in MSE buffer with a polytron-type tissue grinder at 11,000 revolutions per min (rpm) for 2.5 s followed by two rapid strokes at 500 rpm using a loose fit Potter-Elvehjem tissue grinder. Homogenates were centrifuged at 500 × g twice for 5 min, following which supernatants were removed. Mitochondrial pellets were then precipitated twice from supernatants at 3,000 × g, and were subsequently rinsed with MSE buffer. Pellets were then rinsed and resuspended in 50 μL incubation medium containing 220 mM mannitol, 70 mM sucrose, 1 mM EGTA, 5 mM MOPS (pH 7.4), 2 mM taurine, 10 mM MgCl_2_, 5 mM KH_2_PO_4_, and 0.2% BSA. Mitochondria were then incubated for 15 min on wet ice, and protein concentrations were determined using Bradford assays with BSA as a standard. All procedures were performed on wet ice to limit mitochondrial respiratory activity.

### Mitochondrial purification

Three animals from each experimental group were processed for proteomics analyses. Heart tissues were homogenized using a Dounce Tissue Grinder in cold HE buffer containing 10 mM 4-(2-hydroxyethyl)-1-piperazineethanesulfonic acid (HEPES) (pH 7.5), 1 mM EGTA, 0.25 M sucrose and homogenates were then centrifuged twice at 800 × g for 5 min. Supernatants were further centrifuged twice at 4,000 × g for 5 min at 4 °C, and crude pellets were resuspended and submitted to the three-step 18%, 29%, and 52% Percoll-gradient fractionation in 0.3 M sucrose and 10 mM MOPS/KOH (pH 7.2). After 45 min centrifugation at 70,000 × g (Beckman SW 40 rotor, Beckman Coulter), intact mitochondria were isolated from the 29%/52% interphase. Subsequently, iTRAQ proteomics analyses were performed by solubilizing purified mitochondria in Tissue Protein Extraction Reagent (PIERCE). In these experiments, 60 μg protein samples were pooled from each group before proteomic analyses because extensive analysis of well-characterized pooled samples is more productive than analyzing individual samples.

### iTRAQ proteomics

A commercial iTRAQ analysis system (Filgen, Nagoya, Japan) was used with mass spectrometry. Briefly, 100 μg protein samples were reduced and alkylated prior to trypsin digestion, and the resulting peptides were lyophilized and reconstituted before labeling with sham control-iTRAQ 114, TAC 2W-iTRAQ 115, and TAC 8W-iTRAQ 116 according to the manufacturer’s instructions (AB SCIEX). Labeled digests were combined into sample mixtures, and protein identification and relative iTRAQ quantitation were performed using an AB SCIEX TripleTOF 5600 mass spectrometer with ProteinPilot™ software version 4.5 using the Paragon™ Algorithm 4.5.0.0. Electron transport chain (ETC) proteins were identified using the Human Genome Organisation (HUGO) Gene Nomenclature Committee (HGNC) database (http://www.genenames.org/genefamilies/mitocomplex).

### Microarray

Analyses were performed in sham-operated mice (Sham) and TAC-operated mice at 8 weeks (TAC 8W). Total RNA was extracted from hearts using TRIzol reagent (Invitrogen) followed by RNeasy clean-up (Qiagen) according to the manufacturer’s instructions. Total RNA samples from each group were combined into single samples and expression of 36,142 transcripts was profiled by an analysis service (Filgen). Briefly, RNA samples were assessed for RNA integrity on a Bioanalyzer2100 (Agilent Technologies), and target RNA for hybridization was prepared using a MessageAmpTMII-Biotin Enhanced Kit (ambion). Fragmented target RNA (10 μg) was used for hybridization to CodeLinkTM Mouse Whole Genome Bioarray slides (Applied Microarrays). Microarrays were then washed and processed using a direct detection method for biotin-containing transcripts with a streptavidin-Cy5 conjugate. Slides were scanned using a GenePix 4000B laser scanner (Molecular Devices), and images were digitized using CodeLinkTM Expression Analysis v5.0 (Applied Microarrays). Data were normalized and expressed as fold increases relative to data from sham-operated hearts using the MicroArray Data Analysis Tool Ver. 3.2 (Filgen).

### Identification of 9030617O03Rik

To identify novel factors that regulate heart failure, we analyzed changes in the mitochondrial proteome following pressure overload heart failure. In these analyses, heart mitochondria were purified and iTRAQ was used for simultaneous identification and relative quantification of mitochondrial proteins through isobaric peptide tagging. Comparisons were made between Sham and TAC 8W. A total of 7,925 iTRAQ-labeled peptides were mapped to a total of 464 proteins, which were then identified and quantified in purified heart mitochondria. Among these proteins, 9030617O03Rik was recognized as a mitochondrial protein with unknown function, and its expression was decreased in TAC mice.

### Cell culture

H9C2 rat cardiomyoblasts were cultured in Dulbecco’s modified Eagle medium (DMEM) supplemented with 10% fetal bovine serum (FBS), 100 μg mL^−1^ penicillin, and 100 μg mL^−1^ streptomycin. Primary cultures of neonatal rat cardiac myocytes were prepared from neonatal Wistar rat hearts. Briefly, cardiac ventricles from 1-day-old rats were minced and dissociated with 0.2% type I collagenase. Dispersed cells were then incubated in 100 mm culture dishes for 30 min at 37 °C, and nonattached cardiac myocytes were collected and incubated in DMEM supplemented with 10% FBS. Bromodeoxyuridine (1 × 10^−4^ mol/L) was added during the first 48 h to inhibit the proliferation of non-myocytes.

### Plasmids and RNA interference

Nonspecific and 9030617O03Rik-specific small interfering RNA (siRNA) duplexes were purchased from Invitrogen and were transiently transfected using Lipofectamine RNAiMAX (Invitrogen) according to the product protocol.

### Immunoblot

Western blotting analyses were performed with mouse anti-GAPDH (1:2,000, MAB374, Millipore), mouse anti-β actin (1:2,000, AC-74 Sigma), rabbit anti-TOM20 (1:1,000, sc-11415, Santa Cruz), rabbit anti-Tubulin (1:1,000, RB-9281-P0, Thermo), mouse anti-Opa1 (1:500, 612606, BD Biosciences), rabbit anti-GRP75 (1:1,000, sc-13967, Santa Cruz), and mouse anti-NDUFA9 (1:1,000, 459100, Invitrogen) antibodies. Horseradish peroxidase (HRP)-conjugated secondary antibodies were purchased from GE healthcare. Signals on polyvinylidene difluoride (PVDF) membranes (Biorad) were detected using ECL prime (GE healthcare). Anti-9030617O03Rik rabbit polyclonal antibodies were generated against the full length mouse 9030617O03Rik peptide (1:1,000).

### Real-time RT-PCR

Total RNA was isolated from mouse hearts using RNA extraction and nucleic acid purification kits (TAKARA, Otsu, Japan) according to the manufacturer’s protocols. Subsequently, complementary DNAs (cDNAs) were generated using a Prime Script reverse transcription (RT) reagent kit (TAKARA), and real-time PCR was performed using SYBR green (Invitrogen). An initial denaturation step was performed for 10 min at 95 °C and was followed by 45 cycles of amplification at 95 °C for 10 s, 62 °C for 10 s, and 72 °C for 30 s. Gene expression was quantified using β-actin as an internal control with the following primers: mouse 9030617O03Rik, sense 5′-TGTCGGCCCGAGGATGTCCC-3′ and antisense 5′-GGGCTGGCGAAAGCCAATGGA-3′, and mouse β-actin, sense 5′-CCGTGAAAAGATGACCCAGA-3′ and antisense 5′-AGAGGCATACAGGGACAGCA-3′.

### Chemicals

Proteinogenic L-amino acids and their enantiomers, as well as γ-aminobutyric acid were purchased from Sigma-Aldrich (St. Louis, MO, USA). L-Tryptophan methyl ester hydrochloride and 1-(3-dimethylaminopropyl)-3-ethylcarbodiimide hydrochloride were purchased from Tokyo Chemical Industry Co., Ltd. (Tokyo, Japan) and *o*-phthalaldehyde (OPA), Boc-L-cysteine, 5-oxo-D-proline, and 5-oxo-L-proline were purchased from Wako Pure Chemical Industries, Ltd. (Osaka, Japan), Novabiochem (Läufelfingen, Switzerland), Combi-Blocks, Inc. (San Diego, CA, USA), and Enamin Ltd. (Kiev, Ukraine), respectively. All other chemicals were of the highest grade available and were purchased from commercial sources.

## Additional Information

**How to cite this article**: Ariyoshi, M. *et al*. _D_-Glutamate is metabolized in the heart mitochondria. *Sci. Rep.* **7**, 43911; doi: 10.1038/srep43911 (2017).

**Publisher's note:** Springer Nature remains neutral with regard to jurisdictional claims in published maps and institutional affiliations.

## Supplementary Material

Supplementary Information

## Figures and Tables

**Table 1:** Determination of chiral amino acids in 9030617O03Rik (+/+) and (−/−) mouse hearts by two-dimensional high-performance liquid chromatography (2D-HPLC).

	L-analogue		D-analogue	
	+/+	−/−	+/+	−/−
His	896.2 ± 67.1	920.1 ± 106.9	nd	nd
Asn	899.6 ± 97.2	1059.9 ± 246.4	nd	nd
Ser	1572.8 ± 142.8	1272.6 ± 380.4	10.2 ± 2.6	4.2 ± 0.8
Gln	6673.3 ± 224.2	6934.9 ± 354.7	nd	nd
Arg	1440.4 ± 78.5	1082.2 ± 76.7	4.95 ± 1.1	nd
Asp	5618.5 ± 286.0	6216.7 ± 252.3	19.7 ± 0.8	15.2 ± 0.9
Gly	2123.2 ± 26.5	2010.0 ± 81.8	—	—
*allo*-Thr	nd	nd	nd	nd
Glu	26193.9 ± 1296.6	25835.5 ± 1027.8	31.0 ± 1.3	187.3 ± 11.5 *
Thr	1523.2 ± 83.4	1181.8 ± 137.7	nd	nd
Ala	10048.3 ± 603.1	10119.0 ± 630.2	10.2 ± 1.8	3.5 ± 0.4
Pro	460.5 ± 44.4	319.8 ± 25.9	9.0 ± 1.3	5.2 ± 0.9
Met	296.3 ± 25.1	239.9 ± 18.5	nd	nd
Val	759.6 ± 81.2	663.6 ± 16.1	nd	nd
*allo*-Ile	nd	nd	nd	nd
Ile	263.3 ± 22.1	234.5 ± 13.3	nd	nd
Leu	652.0 ± 48.3	529.4 ± 1.6	3.3 ± 1.0	nd
Phe	346.0 ± 19.5	298.5 ± 16.6	nd	nd
Trp	74.8 ± 23.9	44.7 ± 4.7	nd	nd
Lys	1464.7 ± 119.7	1334.9 ± 161.6	nd	nd
Cys	nd	nd	nd	nd
Tyr	475.2 ± 58.8	355.4 ± 40.0	nd	nd

Values are the mean ± S.D. expressed as nmol/g of tissue, obtained by 2D-HPLC analysis from three mice of 13 weeks old, respectively. In addition, “nd” indicates amino acid peaks that were not detected. *p < 0.01.
