# Supplementary material for: D-Glutamate is metabolized in the heart mitochondria
Source: Sci Rep. 2017 Mar 7;7:43911. doi: 10.1038/srep43911 (PMC5339696; doi:10.1038/srep43911)
Supplement: Supplementary Information [file srep43911-s1.pdf]

Supplementary Information

D-Glutamate is metabolized in the heart mitochondria

Makoto Ariyoshi, Masumi Katane, Kenji Hamase, Yurika Miyoshi, Maiko Nakane, Atsushi Hoshino, Yoshifumi Okawa, Yuichiro Mita, Satoshi Kaimoto, Motoki Uchihashi, Kuniyoshi Fukai, Kazunori Ono, Syuhei Tateishi, Daichi Hato, Ryoetsu Yamanaka, Sakiko Honda, Yohei Fushimura, Eri Iwai-Kanai, Naotada Ishihara, Masashi Mita, Hiroshi Homma, Satoaki Matoba

6 Supplementary figures
1 Supplementary table

SI Materials and Methods

Generation of 9030617O03Rik knockout mice

9030617O03Rik knockout mice were created primarily with Knockout Mouse Project (KOMP) targeted ES cells derived from the C57BL/6 mouse strain. The targeting strategy and cell lines produced were described previously[1], and the specific alleles manufacturer's instructions were described in the KOMP Repository (www.komp.org) and the project webpage (www.kompphenotype.org). Using this ES cell (D-10), we mated the mice with CAG-Cre mice to make 9030617O03Rik knockout mice.

Construction of 9030617O03Rik expression plasmids

Mouse 9030617O03Rik was amplified using PCR with the forward and reverse primers 5′-GCCTGGTGCCGCGCGGCAGCCATATGAACACTTCCAGCATGACGGA-3′ and 5′-TCGGGCTTTGTTAGCAGCCGGATCCTCACATGTGCACTGTGGTGG-3′, respectively, to replace the ATG codon of 9030617O03Rik with a *Nde*I restriction site and to add a *Bam*HI restriction site downstream of the Stop codon of 9030617O03Rik. Subsequently, the 9030617O03Rik fragment was reinserted between *Nde*I and *Bam*HI sites of pET-15b (Novagen) using In-Fusion HD cloning Kits (Clontech).

Expression and purification of recombinant protein

Escherichia coli BL21 (DE3) pLysS cells were transformed with the expression plasmid and were cultured at 37°C with shaking in Luria-Bertani medium containing ampicillin (100 μg/mL). Cultures were grown to A_{620} = 0.5 and were then incubated for an additional 30 min at 30°C. After addition of 0.1 mM isopropyl-β-D-thiogalactopyranoside, cultures were incubated at 30°C for a further 16 h, and cells were centrifuged at 10,000 × g for 10 min at 4°C. Subsequently, crude extracts were prepared using BugBuster Protein Extraction Reagent and Lysonase Bioprocessing Reagent (Novagen, Madison, WI, USA) in the presence of protease inhibitors (Nacalai Tesque, Kyoto, Japan) according to the manufacturer's instructions.

Recombinant protein was purified using affinity chromatography with a chelating column. Specifically, crude extracts (prepared as described above) were mixed with 1/49 volumes of 20 mM sodium phosphate buffer (pH 7.4) containing 0.5 M NaCl and 500 mM imidazole and were applied to a His GraviTrap column (GE Healthcare Bio-Sciences Corp., Piscataway, NJ, USA) that had been equilibrated with 20 mM sodium phosphate buffer (pH 7.4) containing 0.5 M NaCl and 10 mM imidazole. The column was then washed with the same buffer, and bound proteins were eluted using 20 mM sodium phosphate buffer (pH 7.4) containing 0.5 M NaCl and 500 mM imidazole. The eluted fraction (6 mL) containing recombinant protein was dialyzed at 4°C for 1 day against 1

L of 50 mM Tris-HCl buffer (pH 8.0) containing 5 mM 2-mercaptoethanol and 10% (v/v) glycerol. The dialyzed fraction was mixed with a solution containing 10 mM phosphate buffered saline (pH 7.4) and 10 mM imidazole in a final volume of 50 mL. Subsequently, the mixture was applied to a His GraviTrap column that had been equilibrated as described above. The column was then washed with 20 mM sodium phosphate buffer (pH 7.4) containing 0.5 M NaCl and 50 mM imidazole, and bound proteins were eluted using a stepwise gradient of 100-500 mM imidazole. Fractions (2 mL) containing recombinant protein were dialyzed at 4°C for 1 day against 1 L of 50 mM Tris-HCl buffer (pH 8.0) containing 5 mM 2-mercaptoethanol and 10% (v/v) glycerol. The buffer was changed once during dialysis, and dialyzed fractions were centrifuged at 10,000 × g for 10 min at 4°C to pellet proteins that were denatured during dialysis. Supernatants were recovered as purified enzyme, were mixed with an equal volume of 100% (v/v) glycerol, and were used immediately for enzyme assays or were stored at –20°C until use.

Purification of the recombinant protein to near -homogeneity was confirmed using SDS-polyacrylamide gel electrophoresis, and protein concentrations of purified enzyme preparations were determined using Bio-Rad Protein Assay Kits (Bio-Rad Laboratories, Hercules, CA, USA) with BSA as a standard.

Enzyme activity assays

Amino acid racemase activity was assayed by measuring L-aspartate, L-glutamate, L-serine, and L-alanine after formation from respective enantiomers in reaction mixtures. Specifically, appropriate amounts (19-37 μg) of purified enzyme were added to reaction mixtures of 50 mM Tris-HCl buffer (pH 8.0), 1 mM dithiothreitol, and 10 mM amino acids in a final volume of 150 μL. Reaction mixtures were then incubated at 37°C for 2 h, and 600 μL aliquots of 100% (v/v) methanol were added to stop the reaction. Subsequently, mixtures were incubated at –80°C for 1 h and centrifuged at 20,000 × g for 10 min at 4°C to remove precipitated proteins. Supernatants (600 μL) were then filtered through a 0.45 μm Millex-LH filter (Millipore, Bedford, MA, USA), and filtrates were diluted appropriately with H_2O. Amino acid analyses were performed as described by Hashimoto *et al.*[2]. Briefly, 10 μL aliquots of diluent were mixed with 30 μL of 400 mM borate buffer (pH 9.0) and 20 μL of OPA/Boc-L-cysteine reagent, which was prepared by mixing 10 mg of OPA with 10 mg of Boc-L-cysteine in 1 mL of 100% [v/v] methanol, and amino acids were derivatized to fluorescent forms in the mixture. After incubation at room temperature for 2 min, 10 μL aliquots were injected into a Jasco chromatographic system comprising a model PU-2089 pump, a model FP-2025 fluorescence detector, and a model 807-IT integrator (Jasco Corp., Tokyo, Japan). Amino acid derivatives were separated on an octadecylsilyl silica gel column (Mightysil RP-18GP, 150 × 4.6 mm internal diameter;

Kanto Chemical Co., Tokyo, Japan) at a flow rate of 0.8 mL/min with a gradient comprising solution A (50 mM sodium acetate buffer, pH 5.9) and solution B (100% [v/v] methanol) as follows: 0 to 25 min, 25 to 35% solution B; 25 to 44 min, 35% solution B; 44 to 45 min, 35 to 37% solution B; 45 to 75 min, 37 to 40% solution B; and 75 to 100 min, 40% solution B. Fluorescence was detected at an excitation wavelength of 344 nm and an emission wavelength of 443 nm. Subsequently, D- and L-amino acid concentrations were determined according to peak areas in chromatograms.

Oxidase and dehydrogenase activities against D-glutamate were determined using a colorimetric method for 2-oxoglutaric acid production as described previously [3]. Briefly, the reaction mixture was prepared as described above and 10 mM D-glutamate was used as the substrate. Mixtures were incubated at 37°C for 2 h, and 10 µL of 100% (w/v) trichloroacetic acid was added to stop the reaction. The produced 2-oxoglutaric acid was reacted with 2,4-dinitrophenylhydrazine and was quantitated by measuring A_{445} against a blank mixture that lacked D-glutamate. The limit of quantitation of this method was 0.019.

Glutamine synthetase and decarboxylase activities were determined by measuring L-glutamine, D-glutamine, and/or γ-aminobutyric acid contents of reaction mixtures. Briefly, reaction mixtures were prepared as described above and 10 mM D-glutamate was

used as a substrate. Mixtures were then incubated at 37°C for 2 h, and 600 µL of 100% (v/v) methanol was added to stop the reaction. Subsequently, mixtures were incubated at –80°C for 1 h and centrifuged at 20,000 × g for 10 min at 4°C to remove precipitated proteins. Supernatants (600 µL) were then filtered through 0.45 µm Millex-LH filters, and filtrates were appropriately diluted with H_2O. Diluents were then applied to high-performance liquid chromatography (HPLC) analyses following OPA/Boc-L-cysteine derivatization as described above.

Glutamate cyclase activity was determined by measuring the reduction of D- or L-glutamate contents in reaction mixtures. Briefly, reaction mixtures were prepared as described above using 10 mM D- or L-glutamate as substrate. Unless otherwise noted, mixtures were incubated at 37°C for 2 h, and then 600 µL of 100% (v/v) methanol was added to stop the reaction. Subsequently, mixtures were incubated at –80°C for 1 h and centrifuged at 20,000 × g for 10 min at 4°C to remove precipitated proteins. Supernatants (600 µL) were then filtered through 0.45-µm Millex-LH filters, and filtrates were appropriately diluted with H_2O. Diluents were then analyzed using HPLC after OPA/Boc-L-cysteine derivatization as described above.

Glutamate cyclase activity was also determined by measuring the formation of D- or L-glutamate from 5-oxo-D- or 5-oxo-L-proline, respectively. Briefly, reaction mixtures were

prepared as described above, and 10 mM 5-oxo-D- or 5-oxo-L-proline were used as substrates. Mixtures were incubated at 37°C for 2 h, and 600-µL aliquots of 100% (v/v) methanol were added to stop the reactions. Subsequently, mixtures were incubated at –80°C for 1 h and centrifuged at 20,000 × g for 10 min at 4°C to remove precipitated proteins. Supernatants (600 µL) were then filtered through 0.45 µm Millex-LH filters, and filtrates were appropriately diluted with H_2O. Diluents were then analyzed using HPLC after OPA/Boc-L-cysteine derivatization as described above.

D-Glutamate cyclase activities were assayed by measuring the formation of 5-oxo-D-proline from D-glutamate in reaction mixtures. Specifically, reaction mixtures were prepared as described above, and 10 mM D-glutamate was used as the substrate. Mixtures were incubated at 37°C for 2 h, and 600 µL of 100% (v/v) methanol was then added to stop the reaction. Subsequently, mixtures were incubated at –80°C for 1 h and centrifuged at 20,000 × g for 10 min at 4°C to remove precipitated proteins. Supernatants (600 µL) were then filtered through 0.45 µm Millex-LH filters, and 100-µL aliquots of filtrates were evaporated to dryness. Subsequently, 65 µL aliquots of 2-propanol were added to residues to dissolve 5-oxo-D-proline but not D-glutamate. Suspensions were then vigorously vortexed for 20 s, followed by sonication in a water bath for 5 min and vigorous vortexing for another 20 s to extract 5-oxo-D-proline products. Suspensions were then

centrifuged at 20,000 × g for 5 min at 4°C, and supernatants (60 μL) were transferred to fresh 1.5 mL microtubes. Original pellets were then vigorously vortexed in 60 μL of 2-propanol again for 20 s to further extract 5-oxo-D-proline products. Suspensions were then centrifuged at 20,000 × g for 5 min at 4°C and supernatants (60 μL) were transferred to fresh 1.5 mL microtubes. The extraction procedure was repeated again, and supernatants were combined. A total of 180 μL of recovered supernatant was then filtered through a 0.45 μm Millex-LH filter, and amino acids were derivatized as described by Mochizuki *et al.*[4]. Briefly, aliquots (80 μL) of supernatant were mixed with 100 μL of 10 mM L-tryptophan methyl ester hydrochloride in acetonitrile/methanol (90:10 v/v) and 20 μL of 50 mM 1-(3-dimethylaminopropyl)-3-ethylcarbodiimide hydrochloride in acetonitrile/methanol (90:10 v/v) to derivatize amino acids prior to separation of enantiomers. After incubation at 65°C for 1 h, solutions were filtered through 0.45 μm Millex-LH filters, and 5 μL aliquots of samples were injected into a Jasco chromatographic system comprising a model PU-2089 pump, a model UV-2075 UV-visible detector, and a model 807-IT integrator (Jasco Corp.). Amino acid derivatives were then separated on an octadecylsilyl silica gel column (Mightysil RP-18GP, 150 × 4.6 mm internal diameter; Kanto Chemical Co.) with isocratic elution at a flow rate of 1 mL/min. The mobile phase comprised 50 mM sodium acetate buffer (pH 4.0): methanol

(73:27 v/v), and spectrophotometric determinations were performed at 278 nm. The specific activity was 113 nmol/min/mg. In this experience, we used 1500 nmol of D-Glu, so degradation of 258 nmol into 5-oxo-D-proline and H_2O was reasonable amount. Quantities of 5-oxo-D-proline were determined according to peak areas in chromatograms.

SI References

1. Skarnes, W.C. et al. A conditional knockout resource for the genome-wide study of mouse gene function. *Nature* **474**, 337-42 (2011).
2. Hashimoto, A. et al. The presence of free D-serine in rat brain. *FEBS Lett* **296**, 33-6 (1992).
3. Katane, M., Seida, Y., Sekine, M., Furuchi, T. & Homma, H. *Caenorhabditis elegans* has two genes encoding functional d-aspartate oxidases. *FEBS J* **274**, 137-49 (2007).
4. Mochizuki, T. et al. Relative quantification of enantiomers of chiral amines by high-throughput LC-ESI-MS/MS using isotopic variants of light and heavy L-pyroglutamic acids as the derivatization reagents. *Anal Chim Acta* **773**, 76-82 (2013).

2100 expert_EukaryoteTotal RNA Nano_2011-10-14_002.xad Page 1 of 1

Assay Class: EukaryoteTotal RNA Nano
Data Path: D:\...0-14\2100 expert_EukaryoteTotal RNA Nano_2011-10-14_002.xad

Created: 10/14/2011 1:06:32 PM
Modified: 10/14/2011 1:34:16 PM

Electropherogram Summary

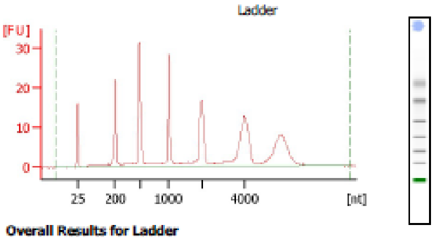

Overall Results for Ladder

RNA Area:	266.2
RNA Concentration:	150 ng/µl
Result Flagging Color:	
Result Flagging Label:	All Other Samples

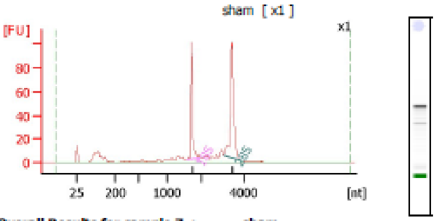

Overall Results for sample 7 : sham

RNA Area:	404.5
RNA Concentration:	228 ng/µl
rRNA Ratio [28s / 18s]:	1.5
RNA Integrity Number (RIN):	9.1 (B.02.05)
Result Flagging Color:	
Result Flagging Label:	RIN: 9.10

Fragment table for sample 7 : sham

Name	Start Size [nt]	End Size [nt]	Area	% of total Area
18S	1,598	1,952	82.9	20.5
28S	3,016	3,883	122.9	30.4

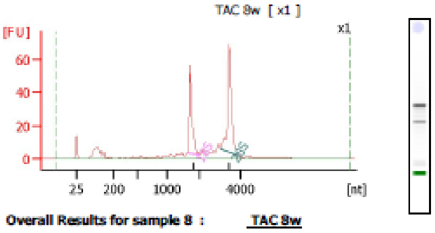

Overall Results for sample 8 : TAC 8w

RNA Area:	340.3
RNA Concentration:	192 ng/µl
rRNA Ratio [28s / 18s]:	1.6
RNA Integrity Number (RIN):	9.2 (B.02.05)
Result Flagging Color:	
Result Flagging Label:	RIN: 9.20

Fragment table for sample 8 : TAC 8w

Name	Start Size [nt]	End Size [nt]	Area	% of total Area
18S	1,659	2,049	62.3	18.3
28S	3,018	3,875	102.7	30.2

2100 expert (B.02.05.SI360) © Copyright 2003-2007 Agilent Technologies, Inc. Printed: 10/14/2011 1:42:04 PM

Fig. S1. QC data of RNA

This data is produced with the consent which is conditioned upon including the following source acknowledgement with each figure, with the year date being the year date shown on the original publication from which the figure was copied: © Agilent 12 Technologies 2003-2007. Reproduced with Permission, Courtesy of Agilent Technologies'

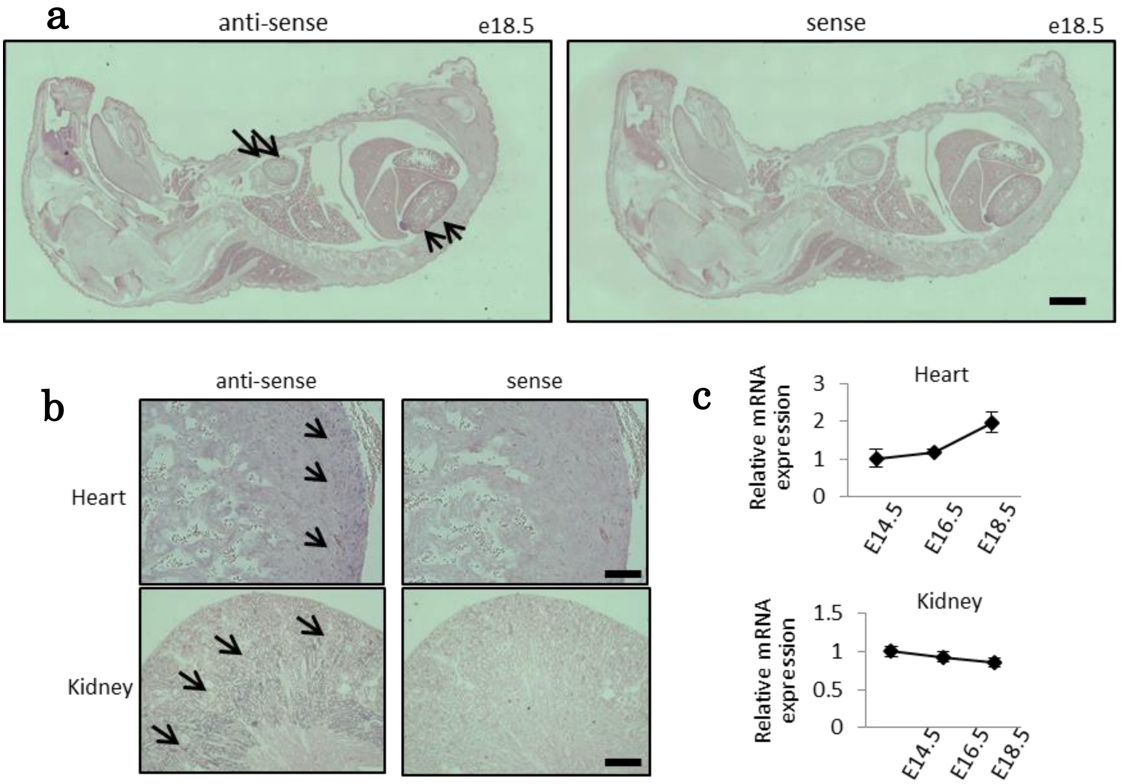

Fig. S2. Expression of 9030617O03Rik during mouse embryogenesis

a and b, Whole-mount *in situ* hybridization of 9030617O03Rik in e18.5 mouse embryos; 9030617O03Rik was expressed in heart and kidney tissues as indicated by arrows. c, Relative expression of 9030617O03Rik in heart and kidney tissues from mouse embryos at indicated embryonic days

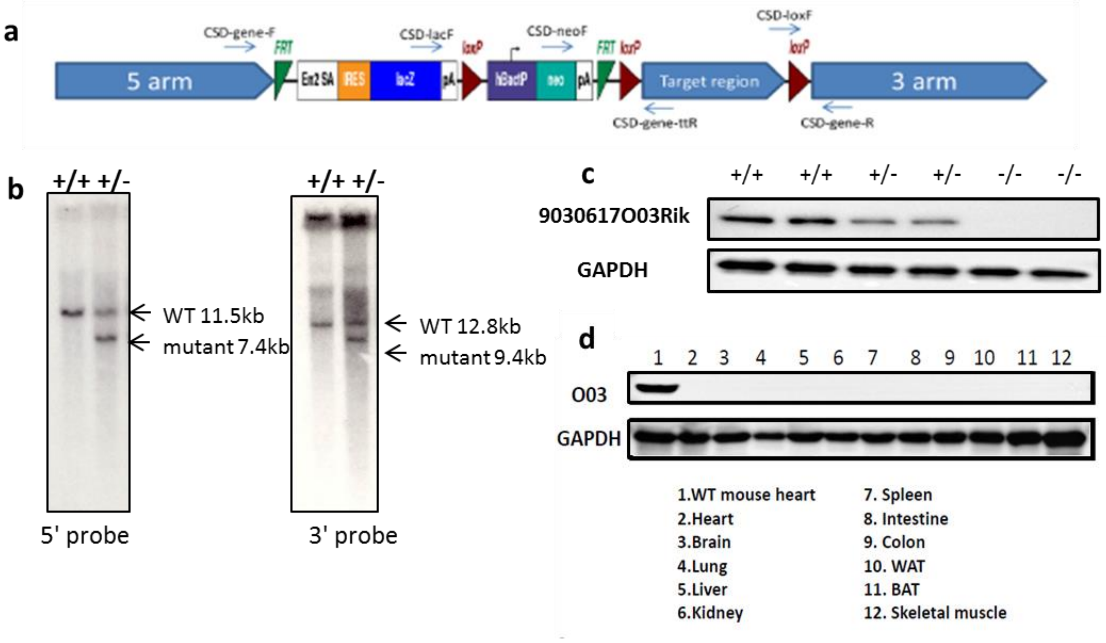

Fig. S3. 9030617O03Rik knock out (KO) mice were generated using an embryonic stem (ES) cell line from the KO mouse project (KOMP) Repository.

a, Schematic representation map of gene arrangements in KO ES cells; b, Representative southern blotting analysis in homo and hetero KO mice; c, Comparison of wild type, hetero, and homo mouse hearts in Western blotting analyses of 9030617O03Rik; d, Western blot analysis of 9030617O03Rik in wild type mouse hearts and several tissues from KO mice.

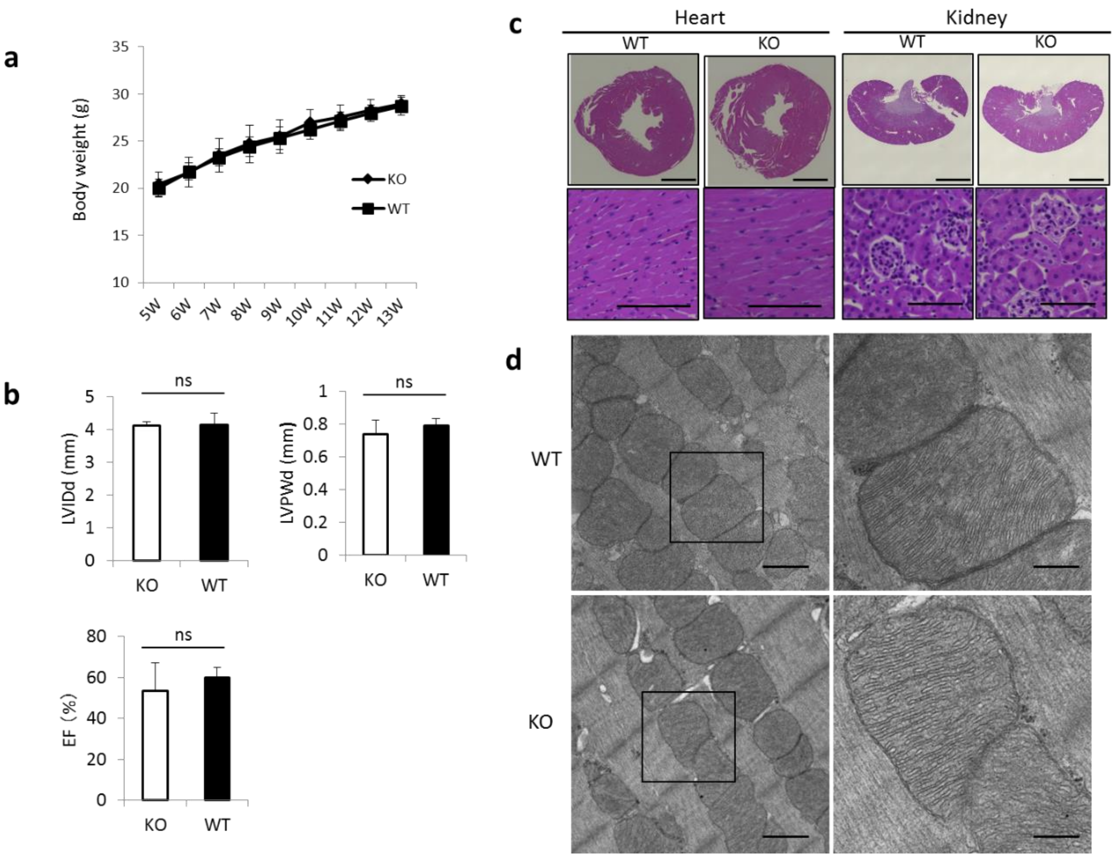

Fig. S4. 9030617O03Rik knock out (KO) mice have no particular phenotype under non stress condition.

a, Body weight. b, Quantitative analyses in echocardiography of diastolic left ventricular dimension (LVDd), left ventricular posterior wall dimension (LVPWd) and ejection fraction (EF). c, Representative images of Hematoxylin-Eosin staining of hearts and kidneys in wild-type (WT) and 9030617O03Rik KO mouse. Higher magnification images are shown (*Lower*); scale bars: 1 mm in *Upper*, and 100 μm in *Lower*. d, Representative electron micrographs of heart; x 2,000; scale bars, 1 μm. Higher magnification images represent mitochondria morphology; x 10,000; scale bars, 200 nm.

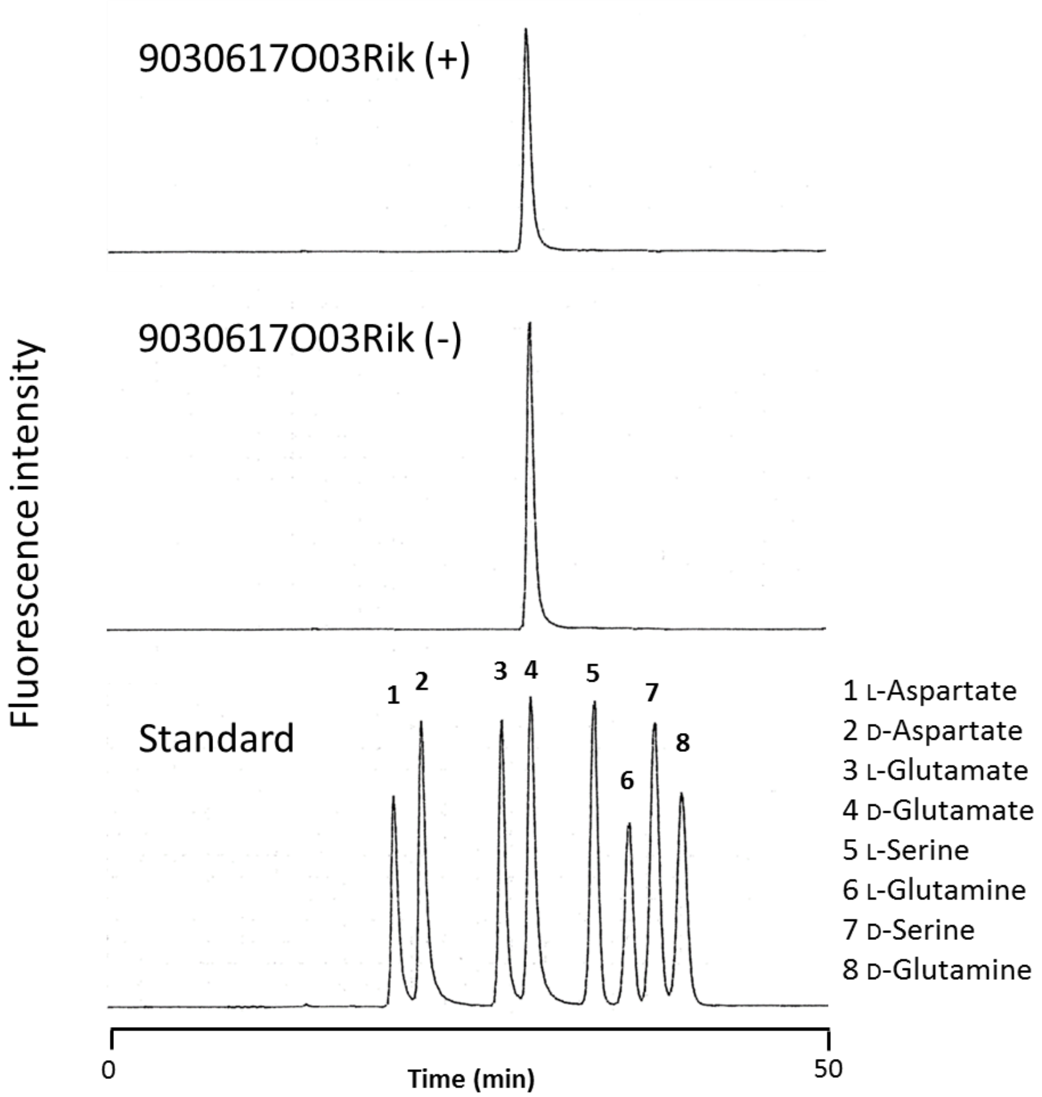

Fig. S5. Enzymatic analysis for glutamate racemase activity

Representative chromatograms of the reaction between D-glutamate and 9030617O03Rik or filtration buffer which was used in purification of 9030617O03Rik as negative control. There was no peak of L-glutamate within both chromatograms. As the standard, 100 pmol each of the indicated amino acids was injected into the HPLC column. These results indicated 9030617O03Rik had no racemase activity. Moreover, the peak reduction of D-glutamate in 9030617O03Rik (+) reaction sample compared to 9030617O03Rik (-) sample also indicated the possibility that D-glutamate was metabolized by 9030617O03Rik as D-glutamate cyclase.

Figure S6. Full-Length blots

Figure 1b

9030617O03Rik

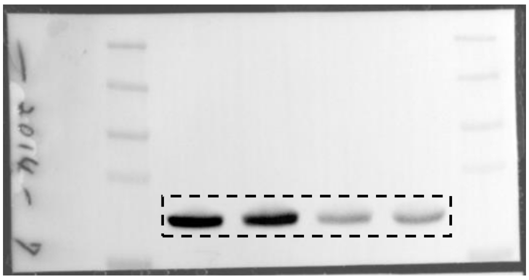

GAPDH

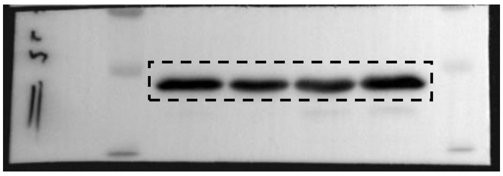

Figure 1d

9030617O03Rik

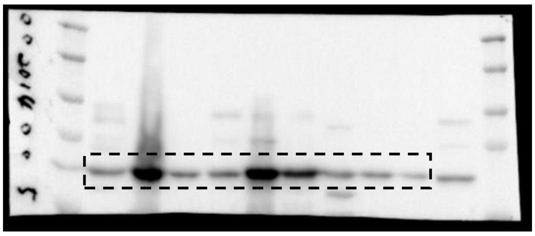

GAPDH

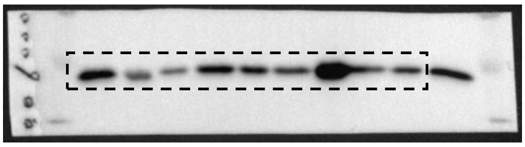

Figure1e

9030617O03Rik

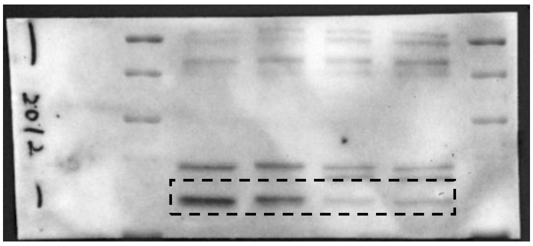

β-actin

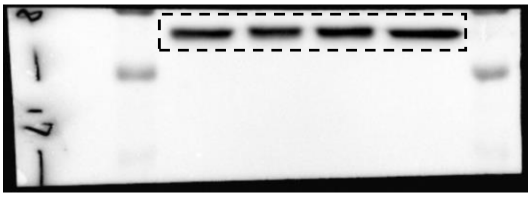

Figure1f

9030617O03Rik

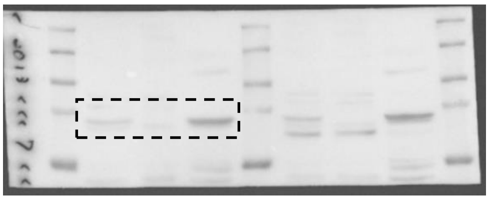

Tom20

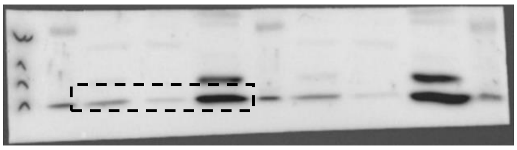

Tublin

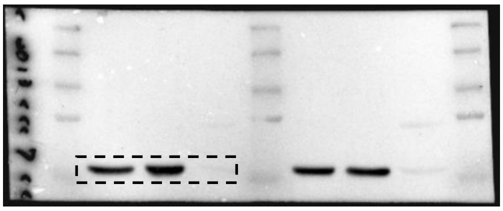

GAPDH

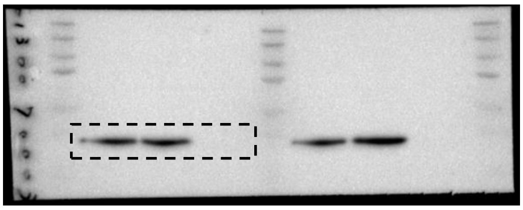

Figure2b

9030617O03Rik

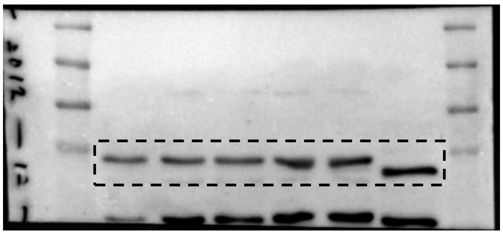

Tom20

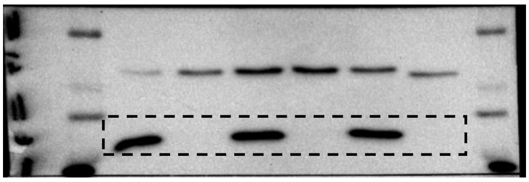

Opa1

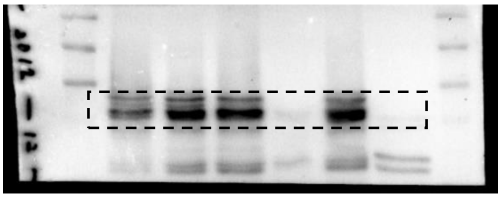

GRP75

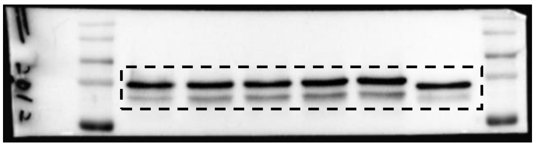

Figure2c

9030617O03Rik

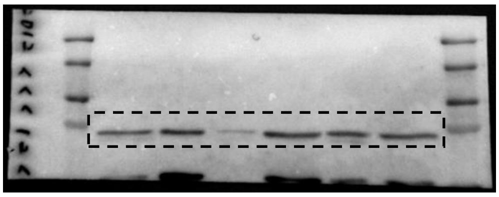

Tom20

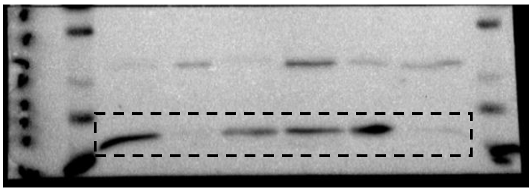

NDUFA9

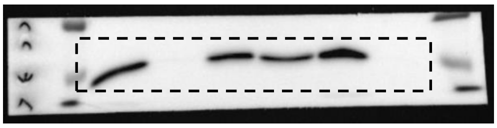

Opa1

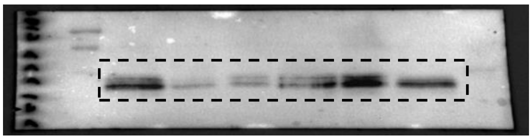

GRP75

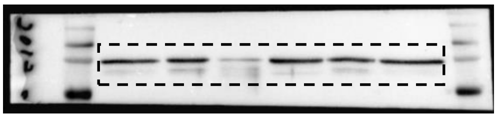

Figure 3f

9030617O03Rik

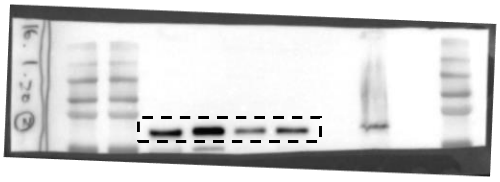

β-actin

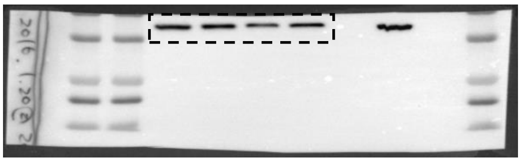

Supplementary information

Figure S2 C

9030617O03Rik

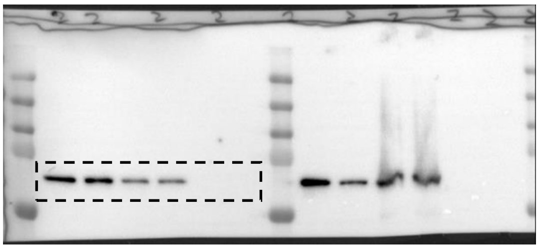

GAPDH

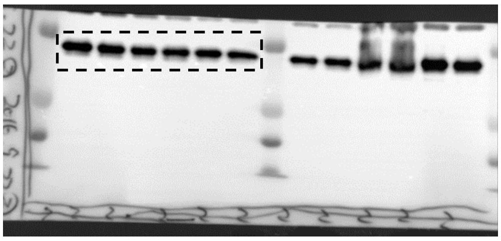

Figure S2 D

9030617O03Rik

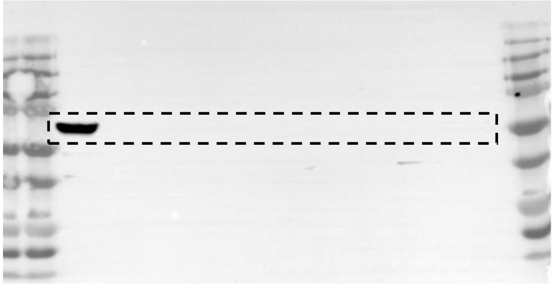

GAPDH

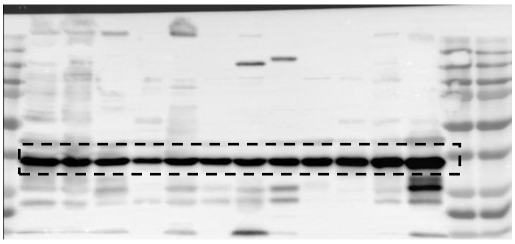

Table S1. Determination of chiral amino acids in 9030617O03Rik (+/+) and (-/-) mouse kidneys by 2D-HPLC

Kidney

(nmol/g)

	L-analogue		D-analogue	
	+/+	-/-	+/+	-/-
His	1056.4 ± 140.1	807.4 ± 16.9	nd	nd
Asn	1705.8 ± 112.5	1577.3 ± 53.8	nd	nd
Ser	4867.7 ± 288.8	4387.3 ± 193.1	41.6 ± 5.7	25.3 ± 1.2
Gln	6673.3 ± 224.2	6934.9 ± 354.7	nd	nd
Arg	2236.3 ± 112.5	1814.2 ± 264.7	24.3 ± 7.5	0.92 ± 0.1
Asp	6270.4 ± 281.1	5907.9 ± 182.2	38.5 ± 2.1	26.0 ± 1.0
Gly	25393.9 ± 994.5	31473.9 ± 1336.3	-	-
allo-Thr	nd	nd	nd	nd
Glu	44927.0 ± 3403.0	52089.5 ± 2743.7	nd	nd
Thr	2657.1 ± 176.6	2167.9 ± 163.8	nd	nd
Ala	6667.4 ± 327.8	6210.0 ± 33.4	nd	82.0 ± 18.3
Pro	2966.1 ± 225.9	2526.4 ± 153.8	nd	nd
Met	1199.8 ± 65.5	1044.2 ± 69.2	6.7 ± 1.7	2.4 ± 0.4
Val	2831.2 ± 325.3	2094.0 ± 100.3	9.0 ± 1.3	5.2 ± 0.9
allo-Ile	nd	nd	nd	nd
Ile	1098.5 ± 74.5	889.7 ± 53.4	nd	nd
Leu	2878.1 ± 187.6	2320.2 ± 129.7	nd	nd
Phe	1372.8 ± 96.3	1122.5 ± 49.9	nd	nd
Trp	220.4 ± 83.9	110.7 ± 3.9	2.4 ± 0.1	nd
Lys	2298.2 ± 185.4	2007.1 ± 103.1	nd	nd
Cys	3162.1 ± 204.9	3893.3 ± 293.0	nd	nd
Tyr	2409 ± 326.5	1757 ± 34.7	nd	Nd

Values are the mean ± S.D. expressed as nmol/g of tissue, obtained by 2D-HPLC analysis from three mice of 13 weeks old, respectively. And ''nd'' indicates amino acid peaks that were not detected. *P<0.01.
